# 

**Published:** 1872-07

**Authors:** 


					﻿)	OUR EXCHANGES.
War Department Medical Circulars.—
We acknowledge the receipt of Circulars Nos.
1, 2 and 3, from the Surgeon General’s Office at
Washington. Circular No. 1 is an elaborate
report upon epidemic cholera and yellow fever
in the U. S. Army during the year 1867. Circu-
lar No, 2 treats of excisions of the head of the
femur for gun-shot injury, elegantly illustrated
with lithographs and wood-cuts of cases. Cir-
cular No. 3 is a large and comprehensive report
upon surgical cases in the army, prepared with
great; pains by Dr. Geo. A. Otis, Ass’t Surgeon,
U. S. A. All these reports are valuable contri-
butions to the medical and surgical literature of
our country.	a
. Philadelphia ■ Medical Times—Continues
to improve in typographical appearance, being
the handsomest medical periodical in the coun-
try, as it is also the best edited.. -
The Medical World, a monthly journal of
medicine and surgery, published by Wm. Bald-
win & Co., 811, Broadway, N. Y., at $1,50 a
year, is a very cheap and excellent journal.
Virginia Clinical Record, a monthly journ-
al of medicine and surgery, published at Rich-
mond, Va., by M. W. Hazlewood, at $1 a year.
Our Southern brethren cannot find a journal
more worthy of their support.
The Northwestern Medical & Surgical
Journal, rather a long name, but equally inter-
esting and valuable journal. It is edited by
Dr. A. J. Stone, monthly, 55 pages, $3 a year,
St, Paul, Minn.
1 •
Detroit Review of Medicine, published at
Detroit, Mich., by Geo. P. Andrews, M. D.,
monthly, 50 pages, $2 a year. Well printed,
edited, and contributed to.
Good Health, published by Alex. Moore,
Boston, monthly, at $2 a year is a popular health
journal, containing health articles from the best
physicians in the land.
Laws of life and Woman’s Health Journal,
edited by Harriet N. Austin, M. D., and publish-
ed at Dansville, N. Y., monthly, at $1,50 a year
has made its appearance in neat magazine
form. We are glad to notice this evidence of
success.
Herald of Health, is one of the best popu-
lar health journals offered to the people. Wood'
& Holbrook, Publishers, 13 & 15 Light-st., N.
Y. Price $2 per annum, with premium.
Peterson, the ever meritorious and popular
ladies’magazine for July, is received. It con-
tains elegant fashion plates, patterns, &c., for
the ladies, and capital stories for all. Chas. J.
Peterson, 306 Chestnut street, Philadelphia, $2 a
year.
American Farmer’s Advocate, is the title of
a new agricultural paper published at Jackson,
rWnn. It is a large quarto of 16 pages, packed,
jffll of valuable reading matter for farmers. It
ns printed on good paper and in fine style, at
only $1 a year. Send for it.
The Lyceum Banner, a child’s paper, pub-
lished at Chicago, every other Saturday, for
only one dollar a year, is much enjoyed by our
children. We therefore notice the fact.
The American Sportsman, a journal devoted
to the interests of the shooting fraternity, and
published by the Parker Brothers, at West
Meridan, Conn., has reached our table. It is an
exceedingly neat eight-page paper, and should
be taken by every sportsman. $1 a year.
The Eye, in Health and Disease, by B. Joy
Jeffries, M. D., of Boston, has been sent us. If
more books of this character were written, it
would be better for thè people. It is an ex-
ceedingly instructive work, published by Alex-
ander Moore, Boston. It is printed upon beau-
tifully tinted paper, and well bound, for $1.50;
OF” We will cause any of the above journals,
to be sent, together with the Bistoury,/?’^, upon
receipt of the publisher’s price. Send the money,
by Post Office order., to our address, and you will
receive any journal selected, together with The
Bistoury, for one year. Of course the War
Department Circulars are not inoluded in this
offer.
Pocket Dictionary.—We have received from
the publishers, 138 and 140 Grand Street, a
copy of Webster’s Pocket Dictionary. It is
neatly printed, bound in morocco, with gilt
edges, and contains 200 pictorial illustrations;
The little volume, while being no larger than
an ordinary pocket book, embraces in its vo-
■cabulary a careful selection of over 18,000 of
the most important words of the language.—
Prefixed to the work are tables of money,
weight and measure, abbreviations, words and
phrases from foreign languages, rules for spell-
ing, explanations, etc. It is in fact a most val-
uable little book, and is doubly worth the
dollar it costs. The publishers, Ivison, Blake-
man & Co., 138 and 140 Grand Street, New
York, will forward it by mail on receipt of
One Dollar, or it can be bought at almost any
book store.
SUBSCRIPTIONS RECEIVED.
In this Column we will credit every subscription re-
ceived during the last quarter. Subscribers will please
notify us immediately if their payments are not duly
credited. The State only is given—this, with the full
name of the party subscribing, will be sufficient to indi-
cate who is meant:
NEW YORK.-T C Cowen, Dr A G Crittenden, C
Rathbun, E Wright, C Rice, C T Stuart, Mrs S Decker,
W V Smith, Dr E P K Smith, II B Knight, S Daws, Wm
'Hartshorn, A B Rice. E T Mathews, Benj Simons, E
Grames, D A Chatfield, W T Felt, E R Thompson, P
Lounsbury, W M Farrar, Jas M Smith, E Miller, A Ab-
bott, R R Hanks, G W Dale, W Morse, C Norris,. T E
Phillips, L E Hewitt, W C Raymond, J D Forest, D D
Bucklin, R Kay, A Tillotson, C. Rice, A H Stephens, A
Carley, Dr D S Miller. M W Lanckton, . J L Cooley, B
Bonner, Dr F A Dudley, Drs Ingersoll & Parkham, Dr P
L Dailey, Dr S R Snook, Dr H M Lahman, Dr S R Mar-
ple, Dr M 0 Dodd, Dr P R Smith, Dr H R Coons, Dr P
Gebart, Dr I S Ketchem, Dr R 0 Fordice, Dr P II Kin-
der, Dr Rowland, Dr JI Keiser, Dr Otto, Dr II Harri-
son, Drs Pollock & Staple, Dr. R S Redfield.
_ PENNSYLVANIA.—Henry Gibbs, G H Wells, Mrs
Mary Braman, Bado Otto, B C Bowman, A Lewis, J II
Wehner, S D Barrows, Miss Lizzie A Roble, J W Nich-
ols, W F Root, R S Sutphin, W T Wrell, Dr W Learning
Mathews, CL Strait, John Finley, Dr E Franciscus, Mrs
J W Chapman, .T C Sanders, Dr M L Bacon, Dr Geo L
Reagan, DrWHWerrick, Dr L M Thompson, Jacob
Brown, Dr G K Weschter, Dr D Macdonnell, Chas A
Hanerstrite, Dr G Albert Smith, Dr G W Moss, Dr N M
Meals, Dr J R Hann, Geo A Cohic, G D Hudson, Dr J E
Blain, J T Learnard. L C Longwell, Drs McKinstry &
Powell, Dr S L Blossom, Dr J McNight, Dr H Pearsoil,
Dr Richter, Dr N Spellinger, Dr P Krautz, Dr MSea-
holtz, Dr W R Reinhart, Dr A Stoffle, Dr Adam Wertz,
Dr 0 M Dailey, Drs Copn & Stough.
ALABAMA.—Col W R R Wyatt, Dr J A Chapin, Mrs
C N Clement, Dr W C Simmons.
CALIFORNIA.—Dr GL Simmons, Mrs IIII Clark.
CANADA WEST.—John L. Davies.
DAKOTA TER.—Jas Smith, Jr.
DIST. COLUMBIA—A Zoller.
GEORGIA.—Dr Chas S Strother.
IOWA.—Dr J Butts, Dr J B Hatton, II H Hemenway.
ILLINOIS.—E E Cornwell.
MISSOURI.—Dr Samuel Morrow, J A Penney.
MICHIGAN.—Frank Ewins, J A Sheldon, H Jolls,
V A Baker, Dr J Vanghon.
MINNESOTA—Samuel Bloomer.
MASSACHUSETTS—Alfred Wood, DrE P Hurd.
MARYLAND.—Dr II B Trist.
NEBRASKA.—C H Whiting.
OHIO.—B J Condit, Dr J Bradley, Drs Reeve & Conk-
lin, Dr S‘R Hamer, Dr G W Pringle.
OREGON.—Dr T N Snow.
SOUTH CAROLINA.—D L Anderson, W AEasterlin.
TENNESSEE.—Jas M Rutledge, Drs Sims A Hamilton.
VERMONT—Chas F Barrett.
VIRGINIA.—Dr N P Trist.
WISCONSIN.—James Johnson.
WYOMING TER.—Margaret McDonald. ,
NO PLACE.—W II Curtis, Thomas Snyder.
NO NAME—Rodney, Vt.
Also, one letter received containing one dollar, with
neither name or place.
The above are the receipts to June 18th. the time of
going to press. Subscriptions received since then will
be credited in next number. ■
TIIE BISTOURY GIVEY AWAY.
OUR TERMS FOR CLUBBING WITH OTHER JOURNALS.
We will cause any of the publications below to be sent
together with The Bistoury, for one year, on the follow-
ing terms:—
PUB. WITH
PRICE. BISTOURY.
American Agriculturalist............81 50	$1	50
Scribner’s Monthly................  4	00	4	00
Peterson’s Ladies’ Magazine......... 2 00	2	00
iHome and Health.................... 1	50	1	50
Little Corporal...................  1	50	1	50
Good Health........................ 2	00	¡2 00
Atlantic Monthly.................... 4	00	4	00
W estern Rural....................  2	00	2	00
Young Folks’ Rural................   1	00	1	00
Peter’s Musical Monthly............ 3	00	3	00
Eclectic Magazine................   5	00	5	00
The Galaxy............-............ 4	00	4 00
Hearth and Home.................... 4	00	4	00
Our Own Friends.....................1	50	1	50
Godey’s Lady’s Book................ 3	00	3	00
Wood’s Household Magazine.'. •..... 1	00	1	00
The Children’s Hour?.................125	125
Saturday Evening Post..........*..... 2 50 •'	2 50
Phrenological Journal..........'.... 3 00	3 00
Medical Times, Philadelphia........ 4 00 ,	4 00
The Aldine ........................ 5 00	5 00
Merry’s Musem........'............. 1	50	1 50
Ballou’s Monthly................... 1 50	1 50
Arthur’s Home Magazine............. 200	200
Monthly Novelet..’................. 2	00	2	00
Our Young Folks.................... 2	00	2	00
Ohio Farmer........................ 2	00	2	00
Cultivator and Country Gentleman... 2 50	2	50
New York Independent............... 2	50	2	50
American Union.. ................   2	50	2	50
Rural New Yorker..-................ 3	00	3	00
Saturday Night .................... 3	00	3	00
Sadies’Repository...-.............  3	50	3	50
Harper’s Magazine.................. 4	00	4	00
Bazar......’.............   4	00	4	00
" Weekly........................ 4	00	4 00
Every Saturday ••••/•*............. 5	00	5	00
North American Review.............  6	00	6	00
Lippincott’s Magazine.............. 4	00	4	00
American Farmer’s Advocate......... 1	00	1	00
Forward the price of any of the above publications to
our address, ana you will receive both journals regularly',
for one year. Address
The Bistoury, Elmira, N. Y.
				

